# Impact of Truncated Oxidized Phosphatidylcholines on Phospholipase A_2_ Activity in Mono- and Polyunsaturated Biomimetic Vesicles

**DOI:** 10.3390/ijms241311166

**Published:** 2023-07-06

**Authors:** Vesela Yordanova, Rusina Hazarosova, Victoria Vitkova, Albena Momchilova, Bozhil Robev, Biliana Nikolova, Plamen Krastev, Philippe Nuss, Miglena I. Angelova, Galya Staneva

**Affiliations:** 1 Institute of Biophysics and Biomedical Engineering, Bulgarian Academy of Sciences, Acad. G. Bonchev Str., Bl. 21, 1113 Sofia, Bulgaria; vyordanova@biophys.bas.bg (V.Y.); r_hazarosova@abv.bg (R.H.); albena_momchilova@abv.bg (A.M.); nikolova@bio21.bas.bg (B.N.); 2 Institute of Solid State Physics, Bulgarian Academy of Sciences, 72 Tzarigradsko Chaussee Blvd., 1784 Sofia, Bulgaria; victoria@issp.bas.bg; 3 Department of Medical Oncology, University Hospital “Sv. Ivan Rilski”, 15 Acad. Ivan Geshov Blvd., 1431 Sofia, Bulgaria; bostro@abv.bg; 4 Cardiology Clinic, University Hospital “St. Ekaterina”, 52 Pencho Slaveikov Blvd., 1431 Sofia, Bulgaria; plamenkr@mail.bg; 5 Centre de Recherche Saint-Antoine, INSERM UMRS 938, Sorbonne Université, 75012 Paris, France; nuss.philippe@gmail.com; 6 Department of Psychiatry, Saint-Antoine Hospital, DMU Neuroscience, Sorbonne University, Assistance Publique-hôpitaux de Paris (AP-HP), 75012 Paris, France; 7 Department of Physics, Faculty of Sciences and Engineering, Sorbonne University, 75005 Paris, France; miglena.anguelova@sorbonne-universite.fr; 8 Matière et Systèmes Complexes (MSC), CNRS UMR 7057, University Paris Cite-Diderot, 75013 Paris, France

**Keywords:** POVPC, PGPC, POPC, PDPC, sPLA_2_ activity, lipid order, vesicle size

## Abstract

The interplay between inflammatory and redox processes is a ubiquitous and critical phenomenon in cell biology that involves numerous biological factors. Among them, secretory phospholipases A_2_ (sPLA_2_) that catalyze the hydrolysis of the *sn-2* ester bond of phospholipids are key players. They can interact or be modulated by the presence of truncated oxidized phosphatidylcholines (OxPCs) produced under oxidative stress from phosphatidylcholine (PC) species. The present study examined this important, but rarely considered, sPLA_2_ modulation induced by the changes in biophysical properties of PC vesicles comprising various OxPC ratios in mono- or poly-unsaturated PCs. Being the most physiologically active OxPCs, 1-palmitoyl-2-(5′-oxo-valeroyl)-*sn*-glycero-3-phosphocholine (POVPC) and 1-palmitoyl-2-glutaryl-*sn*-glycero-3-phosphocholine (PGPC) have been selected for our study. Using fluorescence spectroscopy methods, we compared the effect of OxPCs on the lipid order as well as sPLA_2_ activity in large unilamellar vesicles (LUVs) made of the heteroacid PC, either monounsaturated [1-palmitoyl-2-oleoyl-*sn*-glycero-3-phosphocholine (POPC)], or polyunsaturated [1-palmitoyl-2-docosahexaenoyl-*sn*-glycero-3-phosphocholine (PDPC)] at a physiological temperature. The effect of OxPCs on vesicle size was also assessed in both the mono- and polyunsaturated PC matrices. Results: OxPCs decrease the membrane lipid order of POPC and PDPC mixtures with PGPC inducing a much larger decrease in comparison with POVPC, indicative that the difference takes place at the glycerol level. Compared with POPC, PDPC was able to inhibit sPLA_2_ activity showing a protective effect of PDPC against enzyme hydrolysis. Furthermore, sPLA_2_ activity on its PC substrates was modulated by the OxPC membrane content. POVPC down-regulated sPLA_2_ activity, suggesting anti-inflammatory properties of this truncated oxidized lipid. Interestingly, PGPC had a dual and opposite effect, either inhibitory or enhancing on sPLA_2_ activity, depending on the protocol of lipid mixing. This difference may result from the chemical properties of the shortened *sn-2*-acyl chain residues (aldehyde group for POVPC, and carboxyl for PGPC), being, respectively, zwitterionic or anionic under hydration at physiological conditions.

## 1. Introduction

Remodeling and signaling processes are continuously at play in biological cells. Phospholipases A_2_, in particular the secretory sPLA_2_ family, is a superfamily of functionally diverse enzymes involved in particular in the synthesis of signaling molecules [1] and the remodeling of cell membranes [2,3] via the generation of bioactive lipids and the control of the membrane homeostasis [4]. sPLA_2_ enzymes are involved in the regulation of inflammation and immune responses [5,6] and are one of the many inflammatory biomarkers for oxidative stress-related pathologies, such as diabetes, autoimmune diseases, atherosclerosis, cardiovascular diseases, neurodegenerative disorders or cancers [6,7].

sPLA_2_ are soluble, extracellular, calcium-dependent enzymes characterized by a low molecular weight and His/Asp catalytic dyads in their active site [8,9]. These enzymes catalyze the hydrolysis of the *sn-2* ester bond of the membrane glycerophospholipids (PLs) with the production of free fatty acids (FAs) and lysophospholipids [10]. The physicochemical arrangement of the interface between the enzyme and its membrane substrate modulates the enzyme activity [10,11,12]. Parameters such as the membrane lipid composition, acyl-chain length, degree of FA unsaturation, charge of PL, and the temperature have an impact on the sPLA_2_ activity [13]. The enzyme substrate is embedded in a heterogeneously organized membrane [12,14] in which coexist lamellar liquid–crystalline phases [15]. In cell membranes, relatively packed, ordered phases, enriched in saturated lipid species and cholesterol [16], called liquid-ordered (L_o_) phases, are mixed with liquid-disordered (L_d_) phases [17] mainly composed of unsaturated lipids [18,19].

**Figure 1:**
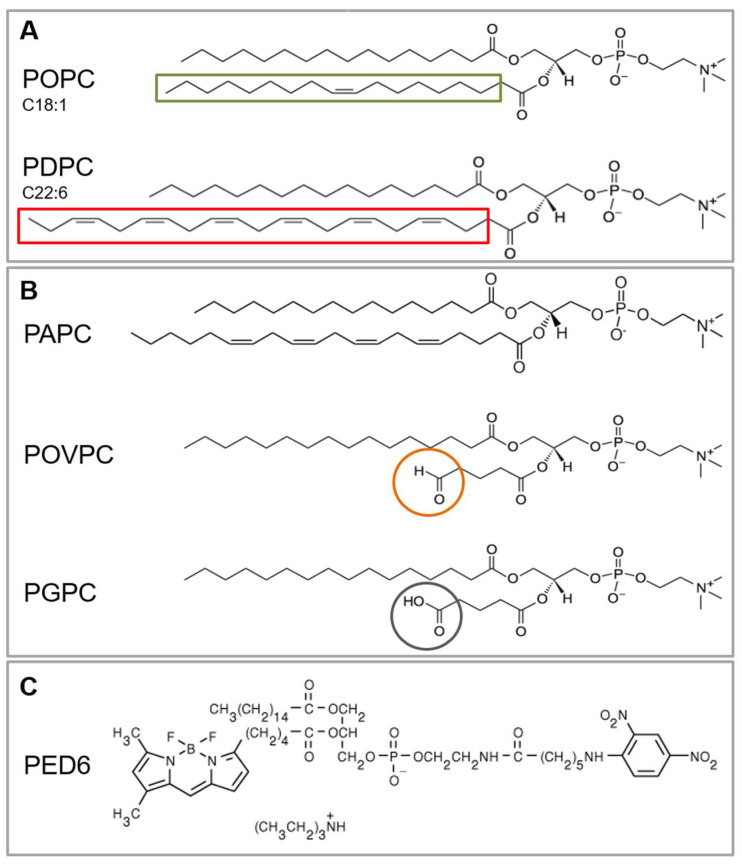
Chemical structures of phospholipids: (**A**) phosphatidylcholine (PC) molecules depending on the degree of fatty acid unsaturation at *sn-2* position: monounsaturated lipid POPC (OA, C18:1) and polyunsaturated one PDPC (DHA, C22:6); (**B**) oxidatively modified phosphatidylcholines differing in the functional group at the free end of the short fatty acid chain at *sn-2* position: POVPC (with aldehyde group) and PGPC (with carboxyl group) and their precursor polyunsaturated lipid PAPC (AA, C20:4); (**C**) glycerophosphoethanolamine lipid analog with BODIPY^®^ dye-labeled *sn-2* acyl chain and dinitrophenyl quencher-modified head group: PED6 (fluorogenic phospholipase A_2_ substrate).

Among polyunsaturated fatty acids (PUFAs) present in the cell membranes, long chain ω-3 PUFAs such as docosahexaenoic acid (DHA) are known to have an important role in human health and diseases [20]. DHA can act as an antioxidant and also exhibits anti-inflammatory properties [21]. Due to their large number of double bonds, PUFA-enriched PLs are susceptible to oxidative damage [22] with the formation of oxidized phospholipids (OxPLs). This lipid peroxidation process is associated with cytotoxicity and apoptosis and plays a significant role in inflammation [23,24]. On one hand, the singlet oxygen is able to dioxidate the tryptophan and open the indole ring, both related to a decrease in sPLA_2_ binding to the lipids and enzyme inactivation [25]. On the other hand, peroxidation significantly affects the physicochemical properties of the membrane lipid bilayers by the formation or reorganization of membrane domains, or specific molecular binding [26]. Lipidomic studies made on pathological tissues could demonstrate that among OxPLs, those containing choline residues were more abundant [23,27]. Two main species, POVPC (1-palmitoyl-2-(5-oxovaleroyl)-*sn*-glycero-3-phosphocholine) and PGPC (1-palmitoyl-2-glutaroyl-*sn*-glycero-3-phosphocholine) (Figure 1B), are able to activate endothelial cells to bind monocytes [28,29]. Moreover, the bioactivity of these compounds has been demonstrated on vascular cells, leukocytes, and platelets [30,31,32,33,34]. POVPC and PGPC were also found in atherosclerotic lesions and are among the most physiologically active oxidized phosphatidylcholines (OxPCs) [26]. Although both OxPCs led to apoptosis, their mechanisms of toxicity are different [35,36,37].

The truncated diacyl phosphatidylcholines POVPC and PGPC are derived from oxidative fragmentation of their precursor PAPC (1-palmitoyl-2-arachidonoyl-*sn*-glycero-3-phosphocholine). They differ only in the nature of the functional group present at the end of the short fatty acid chain in the *sn-2* position, i.e., aldehyde for POVPC, and carboxyl for PGPC (Figure 1B). They are more polar compared to unmodified membrane or lipoprotein phospholipids. Thus, the oxidized groups suggest a lower affinity for the hydrophobic inner part of the bilayer [38] and consequently the possibility to take part in the modifications in fluidity, packing, transition temperature, lateral organization, polarity and permeability.

In the present work, we studied the effect of two OxPCs, POVPC and PGPC (Figure 1B), on vesicle size, membrane lipid order and sPLA_2_ activity, using large unilamellar vesicles (LUVs) presenting a single, homogenous phase (L_d_), made of either monounsaturated (POPC) or polyunsaturated (PDPC) glycerophospholipids (Figure 1A) at physiological salt and temperature conditions. sPLA_2_ activity was assessed by a fluorescence assay based on a fluorogenic phospholipase A substrate, PED6 (Figure 1C).

A major goal of this work is to correlate sPLA_2_ activity with the structural organization of the membranes enriched with polyunsaturated ω-3 lipid (PDPC) and the products of arachidonic acid oxidation, such as POVPC and PGPC.

## 2. Results

### 2.1. PLA_2_ Activity Assay

We performed a sensitive, continuous fluorescence (FL) assay to investigate PLA_2_ activity. PED6-labeled 100% POPC and 100% PDPC LUVs comprising a 10/1 mol/mol PC/PED6 ratio were studied alone and after addition of the POVPC or PGPC. The sPLA_2_ activity was measured in the following lipid mixtures: PC/PED6 100/10 mol/mol (controls), and PC/PED6/OxPC 100/10/30 mol/mol/mol. Of interest, this OxPC addition did not change the PC/PED6 ratio in the mixture, despite the total lipid concentration increasing. Fluorescence changes were monitored in real time after the addition of bee venom sPLA_2_ via the generation of the BODIPY-labeled free fatty acid. FL emission was directly proportional to enzymatic activity and increased as a function of time, following different slopes. The initial rate of the enzymatic by-product was found to be approximately linear for a defined period after the start of the reaction, referred to as the “burst phase”. As the reaction proceeds, the rate continuously slows down, achieving a plateau at a certain time in which the reaction runs at maximum velocity, the so-called steady state. This is a typical enzymatic response generating a hyperbolic curve according to Michaelis–Menten enzymatic kinetics [39,40].

#### 2.1.1. Effect of the Unsaturated Fatty Acid at *sn-2* Position on sPLA_2_ Activity in PC Vesicles

**Figure 2:**
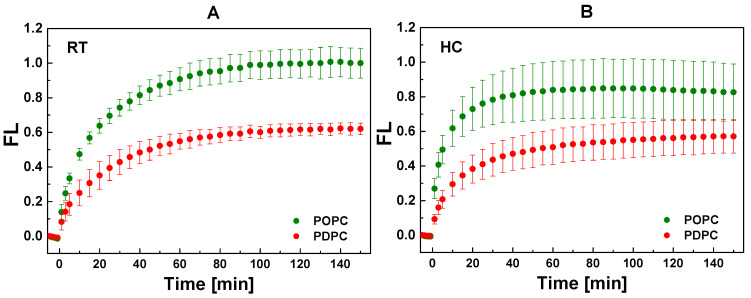
Kinetic curves of PED6 cleavage by sPLA_2_ in POPC and PDPC vesicles. FL = (F_530/_F_530, initial_) − 1 as F_530_ was the fluorescence intensity at 530 nm at time *t* whereas F_530, initial_ represented the sample fluorescence intensity before enzyme addition. Vesicles were formed by hydration at room temperature, RT, (**A**) and by heating/cooling cycles, HC (**B**). sPLA_2_ was added at *t* = 0 min as PC/PED6 and (PC + PED6)/enzyme ratio were 10/1 mol/mol and 8000/1 mol/mol accordingly. FL was read by kinetic setting of 1 min intervals during 2.5 h at 37 °C. The graphs show the FL values measured every 5 min. Error bars corresponded to standard deviations from 12 curves (3 different experiments as each sample is measured 4 times). One-way ANOVA method for means comparison was performed. The data were drawn from a normally distributed population, and the population means were significantly different at the 0.05 level (Supplementary Figure S2).

sPLA_2_ activity kinetics at 37 °C on both POPC and PDPC matrices is presented in Figure 2. One can see that the enzyme activity is generally higher in monounsaturated membranes than in polyunsaturated ones. The FL emission in the monounsaturated matrix was about 2-fold higher in comparison to the polyunsaturated one at t = 10 min for both types of lipid mixing. In the RT-prepared vesicles, the FL value at t = 10 min is almost identical in both types of matrices (47% for POPC vs. 40% for PDPC vesicles), a similarity which is not maintained overtime with a quicker FL increase in POPC vesicles (Figure 2A). Interestingly, for POPC HC-prepared vesicles, 75% of the maximum FL value is reached at t = 10 min, whereas at the same time, only 53% of the maximum FL is obtained for PDPC vesicles (Figure 2B). These results highlight a higher initial rate of enzymatic by-product generation, as well as a more rapidly established steady state for the HC-prepared POPC mixtures.

#### 2.1.2. Effect on sPLA_2_ Activity on POPC and PDPC Vesicles after POVPC or PGPC Addition

**Figure 3:**
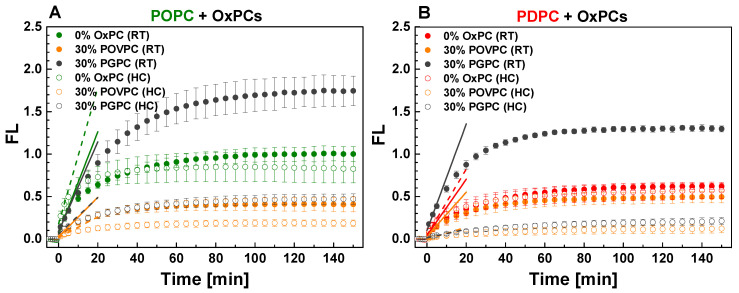
Kinetic curves of PED6 cleavage by sPLA_2_ as PED6 was incorporated into POPC and POPC/OxPC (**A**) as well as PDPC and PDPC/OxPC (**B**) vesicles. FL = (F_530/_F_530, initial_) − 1 as F_530_ was the fluorescence intensity at 530 nm at time *t* whereas *F*_530, initial_ represented the sample fluorescence intensity before enzyme addition. Vesicles were formed by hydration at room temperature (RT) and by heating/cooling cycles (HC). sPLA_2_ was added at *t* = 0 min as PC/PED6 and (PC + PED6)/enzyme ratio are 10:1 mol/mol and 8000:1 mol/mol accordingly. FL signal increase was correspondent to sPLA_2_ activity elevation. FL was read by kinetic setting of 1 min intervals for 2.5 h at 37 °C. On the graphs, FL values of every 5 min were presented for clarity. Error bars corresponded to standard deviations from 12 curves (3 different experiments as each sample is measured 4 times). One-way ANOVA method for means comparison was performed Figure S3). The lines presented linear extrapolations of the initial linear part of the normalized kinetic curves: solid lines for vesicles hydrated at RT and dashed ones for vesicles hydrated with HC cycles. The slopes corresponded to the enzymatic reaction rates.

The kinetics of sPLA_2_ activity in POPC and PDPC LUVs upon POVPC or PGPC addition at 0 and 30 mol % are presented in Figure 3. sPLA_2_ activity was decreased in both lipid matrices after POVPC addition regardless of the type of lipid mixing protocol used. In contrast, the effect of PGPC on enzyme activity differed according to the lipid mixing protocol used. In RT samples, sPLA_2_ activity was higher compared to control for both lipid matrices (Figure 3) while the opposite was observed for HC-prepared samples in both lipid matrices compared to control.

**Figure 4:**
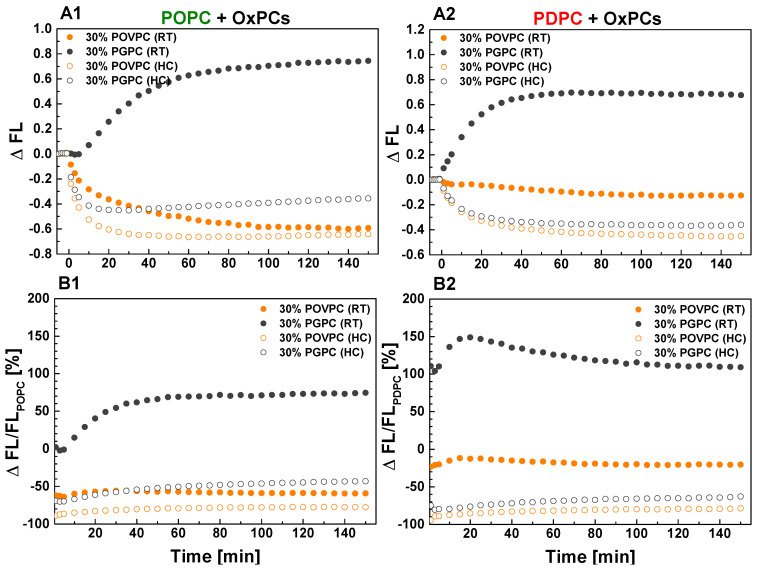
Effect of OxPCs on sPLA_2_ activity in PED6-labeled POPC (**A1**,**B1**) and PDPC (**A2**,**B2**) vesicles presented as absolute (**A**) and relative (**B**) changes. ΔFL was defined as the difference between FL intensity for OxPC-containing vesicles and control PC ones (FL_PC (POPC or PDPC)_). The relative change of FL, ΔFL/FL_PC_ was expressed in percentages for POPC (**B1**) and for PDPC (**B2**). RT—room temperature protocol for lipid mixing; HC—heating/cooling cycles for lipid mixing.

To quantify the effect of OxPCs on sPLA_2_ activity in monounsaturated and polyunsaturated matrices, we calculated absolute and relative changes in FL (Figure 4). The absolute change of FL, ΔFL, was defined as the difference between FL intensity of OxPC-containing vesicles and control PC ones (for example ΔFL_POPC_ = FL_POPC_/_POVPC_ − FL_POPC_) (Figure 4(A1,A2)). The relative change of FL, ΔFL/FL_PC_, was obtained by dividing ΔFL by FL of the control PC (POPC or PDPC) and was expressed in percentages (Figure 4(B1,B2)). As shown in Figure 4B, 30 mol % POVPC in both lipid matrices showed a stronger inhibitory effect on sPLA_2_ activity in HC-prepared samples compared to RT-prepared ones. Furthermore, the reduction in sPLA_2_ activity was less pronounced in PDPC samples with added POVPC than when the same POVPC was introduced into the POPC matrix. Unexpectedly, in RT-prepared samples, the addition of PGPC leads to a more significant increase in sPLA_2_ activity when introduced in a PDPC matrix than in a POPC one. The opposite effect was observed for HC-prepared samples, where enzyme inhibition was again greater when PGPC was added to the PDPC matrix than to the POPC one. For clarity, Table 1 shows the summarized relative ΔFL/FL_PC_ changes of sPLA_2_ hydrolysis in PC/OxPC mixtures at a steady state.

**Figure 5:**
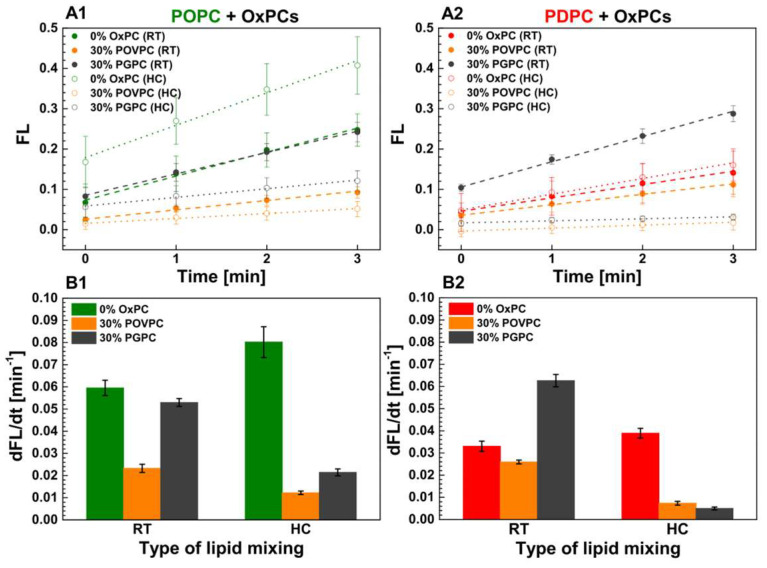
Linear regression of the initial part of the kinetic curves of normalized fluorescence intensity, FL, at 530 nm [FL = F_530_/F_530, initial_ − 1] = *a* + *bt*, where *a* and *b* denoted the y-intercept and the slope, respectively. The intercept yielded the value of the normalized fluorescence intensity FL = F_530_/F_530, initial_ − 1 at *t* = 0 min (**A1**,**A2**). The enzymatic reaction rate was determined as the slope of the kinetic curves (dFL/dt [min^−1^]) (**B1**,**B2**). RT—room temperature protocol for lipid mixing; HC—heating/cooling cycles for lipid mixing.

As mentioned above, the burst reaction of sPLA_2_ hydrolysis is characterized by the initial rate of the enzymatic reaction, which is linear during the first few minutes of the reaction. To assess the rate of sPLA_2_ activity for each studied lipid composition, we analyzed the initial linear part of the normalized kinetic curves (Figure 5, Table 2). The linear regression of the initial part of the kinetic curves of normalized fluorescence intensity (FL) at 530 nm is presented in Figure 5(A1,A2) for both types of the studied binary mixtures with FL = F_530_/F_530, initial_ − 1 = *a* + *bt*, where a and b denote the y-intercept and slope, respectively. Note that fluorescence kinetics reading starts after the enzyme has been aliquoted into the wells, followed by gentle shaking for 20 s. These preliminary steps explain why the initial normalized fluorescence intensity differs from 0 (Figure 5(A1,A2)). Based on the initial slopes of the enzymatic kinetics curves, it can be seen (Figure 5B, Table 2) that, for both types of control PC vesicles, the reaction rate is higher for samples prepared by HC than at RT. The enzymatic reaction rate is highest for POPC control membranes (Figure 5B). This rate is twice as high as that found for PDPC control samples (Table 2). Furthermore, in OxPC-containing mixtures, the enzymatic reaction rate is higher in vesicles prepared at RT than in those prepared by HC.

### 2.2. Effect of POVPC and PGPC on Membrane Lipid Order in POPC or PDPC Lipid Matrix

**Figure 6:**
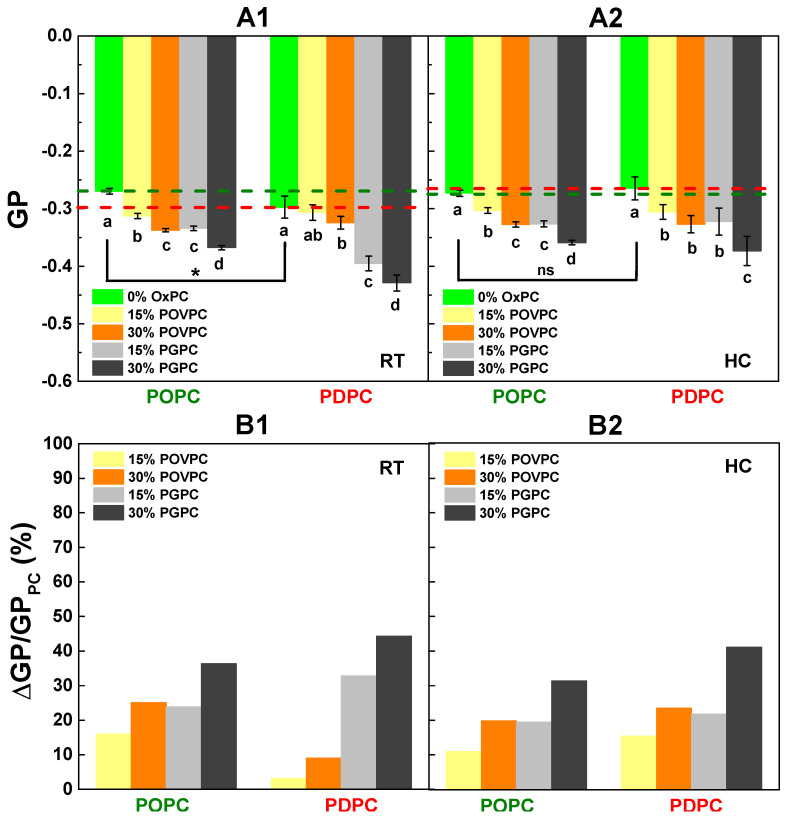
Effect of the degree of FA unsaturation at *sn-2* position of PCs (POPC and PDPC) and OxPC (POVPC or PGPC) on lipid order in PC and PC/OxPC LUVs measured by Laurdan spectroscopy: (**A**) Laurdan GP in control POPC (green dashed line) and PDPC (red dashed line) LUVs, and in binary OxPC-containing (15 and 30 mol %) ones with lipid/Laurdan ratio 200/1 mol/mol hydrated at room temperature, RT, (**A1**), and by heating/cooling cycles, HC, (**A2**). The lines are added only for clarity. The data represented the means of 9 measurements at 37 °C. Error bars corresponded to the standard deviations. One-way ANOVA method for means comparison was performed. The data were drawn from a normally distributed population and showed statistically significant difference in GP values between pure POPC and PDPC vesicles hydrated at RT, based on Tukey test with *p* < 0.05 *, (**A1**). No statistically significant differences (ns) in GP values between POPC and PDPC vesicles hydrated by HC cycles were observed (**A2**). Different letters below bars indicated the statistically significant differences between LUV compositions at both studied protocols of lipid mixing. (**B**) ΔGP/GP_PC_ (%) quantified the reduction in membrane lipid order in presence of OxPC compared to control vesicles without OxPC. RT mixing (**B1**) and HC mixing (**B2**). ΔGP was defined as the difference between averaged GP values for binary OxPC-containing vesicles and control PC ones (GP_PC_).

The effect of OxPCs (POVPC or PGPC) on the lipid order in membrane bilayers was studied in either monounsaturated (POPC) or polyunsaturated (PDPC) PC mixtures using various concentrations of OxPCs. The examined binary LUVs were composed of 85/15 and 70/30 mol/mol PC/OxPC mixtures. Laurdan spectroscopy method allowed identification of the generalized polarization (GP) values of each sample, an indirect quantitative measure of the membrane lipid order. The absolute GP values for pure POPC, PDPC, and binary PC/POVPC and PC/PGPC LUVs are shown on Figure 6A. The relative change of GP values in percentages is also given (Figure 6B). Results from the room temperature (RT) protocol are described on the A1 and B1 columns of Figure 6, while data from the heating/cooling (HC) samples are shown in the A2 and B2 columns of Figure 6. Laurdan GP values for control POPC and PDPC samples were negative, approximating −1.0, which non-surprisingly describes lipids in the L_d_ phase [41]. The higher POPC GP value (−0.270) is indicative of a 10% more compact L_d_ membrane for POPC LUVs compared to PDPC ones (GP = −0.297) (Figure 6(A1)). In pure PDPC LUVs, samples prepared using the HC protocol (Figure 6(A2)) showed an 11% increase in their GP value (−0.265) compared to their RT counterparts (−0.297) (Figure 6(A1)). This increase in GP value removed the difference in lipid order between both studied PC species (Figure 6(A2)).

In binary PC/OxPC mixtures, GP values decreased upon OxPC addition in both RT and HC samples, showing a proportional lipid order decrease with the OxPC increase. PGPC decreased lipid order to a greater extent than POVPC in both PC lipid matrices (Figure 6B). This effect was more pronounced for PDPC containing LUVs. Interestingly, in the PDPC/POVPC binary mixtures, the decrease in lipid order was greater on samples prepared by HC protocol (Figure 6(B2)) than on those prepared by RT (Figure 6(B1)). Such a result shows the importance of sample preparation procedures for order parameter measurements in polyunsaturated lipid mixtures.

### 2.3. Effect of POVPC and PGPC on Vesicle Size in POPC and PDPC Lipid Matrix

In an attempt to understand why the effect of OxPCs on sPLA_2_ activity depends on the lipid mixing protocol, and considering that the binding of the enzyme to its substrate, and therefore its activity, depends on the physicochemical properties of the interface between the enzyme and membrane phospholipids, we decided to measure the mean LUV size with and without OxPC. sPLA_2_ activity depends on how the substrate is exposed to the enzyme. Substrate accessibility varies according to whether it is contained in vesicles or micelles, whose size will modulate the enzymatic activity.

**Figure 7:**
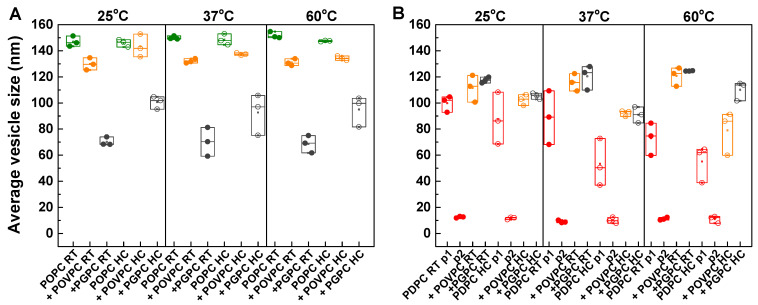
Average vesicle size of large unilamellar vesicles (LUVs) composed of PC/OxPC mixtures as PC is POPC ((**A**), green points) or PDPC ((**B**), red points) whereas OxPC is POVPC (orange points) or PGPC (dark gray points). Two protocols of lipid mixing were used to form LUVs: RT (filled circles) was lipid mixing at room temperature (25 °C) and HC (open circles) was lipid mixing through heating/cooling cycles. Three independent vesicle size experiments were carried out (vesicle preparations) as each point on the graph averaged three measurements from one experiment.

Figure 7 presents the changes in average LUV size induced by the degree of PC unsaturation and type of OxPC (A for POPC and B for PDPC). The size of the LUVs was measured at three different temperatures (25°, 37° and 60 °C). POPC formed LUVs with a larger average size compared to PDPC, as one peak distribution was observed for POPC centered around 145 nm (A) compared to PDPC, where two peaks are seen (B), one centered at 100 nm (p_1_) and the other at about 10 nm (p_2_) at 25 °C.

The lipid mixing protocol and increasing measurement temperature had no impact on the average size of POPC LUVs. On the other hand, for PDPC, mixing protocol and temperature had an impact on LUV size, which ranged from 100 to 50 nm (p_1_). The higher the temperature, the smaller the LUV size. Average vesicle size is reduced under HC preparation compared with RT conditions. This effect is even more pronounced at higher temperatures.

The addition of OxPC to POPC membranes led to a decrease in the mean vesicle size, this decrease being much greater for PGPC (around 40%) than for POVPC (10%) at 25 °C. This decrease in vesicle size was also greater for the RT protocol (50%) than for the HC (30%). In contrast to these results with POPC, the addition of OxPC to PDPC membranes was associated with an increase in the mean vesicle size, with similar increases for POVPC and PGPC. The higher the temperature in the PDPC mixture, the greater the difference in the mean LUV size for the two OxPCs studied. This increase in vesicle size was slightly smaller when the lipids were mixed by HC than RT for both OxPCs.

In summary, these experiments clearly showed that LUV size was more sensitive to the lipid mixing protocol for PDPC mixtures than for POPC ones. For POPC LUVs, the addition of PGPC led to a significant decrease in their size when compared to the addition of POVPC. The lipid mixing protocol also influences LUV size for POPC/PGPC mixtures. An opposite effect is observed on PDPC membranes, where both OxPCs induced a similar increase in vesicle size.

## 3. Materials and Methods

### 3.1. Materials

Lipids: 1-palmitoyl-2-oleoyl-*sn*-glycero-3-phosphocholine (POPC), 1-palmitoyl-2-docosahexaenoyl-*sn*-glycero-3-phosphocholine (PDPC), 1-palmitoyl-2-(5′-oxo-valeroyl)-*sn*-glycero-3-phosphocholine (POVPC), 1-palmitoyl-2-glutaryl-*sn*-glycero-3-phosphocholine (PGPC), all synthetic, were purchased from Avanti Polar Lipids, Inc. (Alabaster, AL, USA) and used without further purification. The fluorescent probe 6-dodecanoyl-N, N-dimethyl-2-naphthylamine (Laurdan) and the phospholipase A_2_ from European honey bee (*Apis mellifera*) venom (bvPLA_2_) were ordered from Sigma-Aldrich (St. Louis, MO, USA). The fluorogenic phospholipase A_2_ substrate N-((6-(2,4-dinitrophenyl) amino) hexanoyl)-2-(4,4-difluoro-5,7-dimethyl-4-bora-3a,4a-diaza-s-indacene-3-pentanoyl)-1-hexadecanoyl-*sn*-glycero-3-phosphoethanolamine, and triethylammonium salt (PED6) were obtained from ThermoFisher Scientific (Waltham, MA, USA).

### 3.2. Liposome Preparation

Samples were prepared by mixing the indicated lipids from stock solutions in chloroform/methanol 1/1 (*v/v*) to obtain the desired compositions in glass tubes. The fluorescent marker Laurdan was dissolved in chloroform/methanol 9/1 (*v/v*) at 0.25 mg/mL concentration. PED6 stock solution (5 mM) in dimethyl sulfoxide (DMSO) was used to prepare PED6 (1 mM) in absolute ethanol for further mixing with lipid solutions. Laurdan and PED6 were mixed with the lipids in the initial organic solutions. Solvents were then evaporated under a gentle stream of oxygen-free dry nitrogen. The tubes were kept overnight under vacuum to remove solvent traces. Tris buffer for lipid hydration (10 mM Tris-HCl, 150 mM NaCl, 0.1 mM CaCl_2_.2H_2_O at pH 7.5 prepared from distilled water) was purged with a stream of oxygen-free nitrogen and then added to the dried lipid films. Aqueous solutions of multilamellar vesicles (MLVs) were prepared using two different protocols (room temperature and heating/cooling) for lipid hydration and mixing. In the room temperature protocol (RT), the buffer, thermostated to room temperature (25 °C), was added to the dried lipid film, also thermostated to room temperature. Then, the sample was vortexed (30 s) and sonicated (30 s) 3 times in ultrasonic bath Fisherbrand^®^ at room temperature. In the heating and cooling cycle protocol (HC), the preheated buffer (60 °C) was added to the dried lipid film at room temperature. Then, the sample was thermostated at 60 °C (5 min), vortexed (30 s), sonicated (30 s) and ice-cooled (5 min) for lipid mixing. HC is widely used and accepted as a classical protocol to ensure ideal lipid mixing in case of lipid mixtures as well as maximum yield of unilamellar vesicles during the latter extrusion step. These operations were repeated 3 times. The “golden standard” to ensure the mixing of two liquid phases and the uniformity of the vesicle composition is to perform the lipid hydration at 60 °C. In our conditions, as the phase transition temperatures of POPC and PDPC are very low, below 0 °C, and not more than 30 mol % OxPCs were added to the main lipids (POPC or PDPC), one cannot expect to observe L_β_/L_d_ phase separation at physiological temperature. Since we do not have any information about the miscibility temperature of POPC or PDPC with POVPC or PGPC, we designed to compare LUV samples hydrated through two different protocols: lipid mixing performed at room temperature (RT) and with heating/cooling (HC) cycles.

MLV suspensions, obtained either by RT or by HC protocol, were further used for preparing large unilamellar vesicles (LUVs) by the extrusion method using a LiposoFast small-volume extruder as follows: 11 passages through polycarbonate filters (Avestin, Ottawa, ON, Canada) with a pore diameter of 800 nm and 21 passages through 100 nm filters. All samples were examined on the same day for the various experiments.

### 3.3. Fluorescence Spectroscopy

Laurdan fluorescence measurements. The fluorescent marker Laurdan is an amphiphilic molecule that detects changes in the phase properties of the membrane through its sensitivity to the polarity of the bilayer. The probe shows specific emission peaks at 440 and 490 nm that originate from lipid membranes in ordered phase (liquid-ordered L_o_ or gel L_β_) and liquid-disordered phase (L_d_), respectively, due to differences in the polarity and in the degree of dipolar relaxation. The generalized polarization (GP), which is a relative quantitative measure for membrane lipid order [42], can be calculated as follows

GP = (I_440_ − I_490_)/(I_440_ + I_490_)

where I_440_ and I_490_ are the emission intensities at 440 and 490 nm. Laurdan GP provides information about the hydration/lipid packing in the space of polar head groups near glycerol backbone. Theoretically, the values for the GP function go from −1.0 (being least ordered) to +1.0 (being most ordered); however, experimentally they range from −0.3 to 0.6 [41] for both pure lipids and mixtures.

Fluorescence (FL) measurements were carried out with a F-7000 spectrofluorometer (Hitachi) equipped with a Xenon arc lamp at 37 °C. A quartz cuvette was used. The final lipid concentration in the cuvette was 500 µM. The temperature in the cuvette holder was maintained using a Julabo thermostat. The excitation wavelength for Laurdan was 355 nm. The emission spectra were recorded from 390 to 600 nm. Samples were run in triplicate. Analysis of the steady-state spectra was performed by using OriginPro 9.0.

Fluorogenic PLA_2_ assay. The PLA_2_ substrate PED6 is a glycerophosphoethanolamine lipid analog with BODIPY^®^ dye-labeled *sn-2* acyl chain and dinitrophenyl quencher-modified head group (Figure 1C). The close proximity of the fluorescent dye to the quencher group prevents FL emission, but liberation of the dye-labeled chain by sPLA_2_ eliminates the intramolecular quenching effect of the dinitrophenyl group, converting the quenched substrate to a fluorescent product, resulting in a corresponding BODIPY FL increase [43,44].

FL measurements were taken with a Synergy^TM^2 microplate reader (BioTek). Additionally, 96-well plates (Greiner) were used. The liposome mixtures (50 μM PC concentration) were aliquoted into 192 µL volumes and measurements were set for kinetic readings (sensitivity = 65) and 1 min time intervals. The well plate was incubated at 37 °C for 10 min, shaken for 20 s and fluorescence was read for 5 min at 485 (±20) nm excitation and 528 (±20) nm emissions. The obtained FL emission intensities represent the baseline recorded for 5 min before PLA_2_ addition as a no-enzyme control. Stock solution of sPLA_2_ (100 µg/mL) from bee venom was stored at 4 °C in Tris buffer as the same buffer was used for lipid hydration and mixing. To be activated the enzyme, sPLA_2_ was first transferred to the assay buffer (10 mM Tris-HCl, 150 mM NaCl, 1 mM CaCl_2_.2H_2_O at pH 7.5) just before being added to the samples for measurements. Then, 8 μL of this sPLA_2_ (2.5 μg/mL) preparation was added to each well for a final volume of 200 μL. The specific activity of this enzyme, provided by Sigma-Aldrich, is 1956.57 units/mg of protein. Considering a molecular weight of sPLA_2_ ~ 14,500 Da, sPLA_2_ concentration of 0.2 units/mL corresponds to 0.1 μg/mL per well. The final substrate (PC+PED6)/enzyme ratio is thus around 8000/1 mol/mol. The well plate was shaken for 20s, and fluorescence activity kinetics were recorded for 2.5 h, starting as soon as possible after enzyme addition. The data were plotted in OriginPro 9.0 to assess changes in fluorescence intensity as a function of time. Due to differences in relative fluorescent units (RFU) of each sample, the kinetic PED6 hydrolysis curves of the POPC samples were normalized to 1.0 at 150 min time point for the RT samples. The curves of the HC samples were recalculated and normalized according to the sPLA_2_ kinetics of the pure POPC samples.

### 3.4. Dynamic Light Scattering (DLS) Measurements

A Zetasizer Nano ZS analyzer (Malvern Instruments, Malvern, UK) with a temperature-controlled cuvette holder was used to perform dynamic light scattering (DLS) measurements to characterize the vesicle size distribution. The samples with 200 μM lipid concentration were run in ZEN1002 universal dip cells (Malvern). The methodology of these measurements is described in detail in Vitkova et al. [45].

## 4. Discussion

Our work investigated the influence of two OxPC lipids (POVPC and PGPC) on sPLA_2_ activity at physiological temperature, where OxPCs were added to a mono- or polyunsaturated PC matrix (POPC or PDPC).

Due to their low phase transition temperatures, POPC (−2 °C) and PDPC (−27 °C) form L_d_-lipid arrangements at physiological temperatures. In contrast to them, no mixing/demixing temperatures are known for studied OxPCs with these glycerophospholipids. Both OxPCs used in the present study contain a 16-carbon, saturated fatty acyl chain (palmitic acid) at the *sn-1* position and differ only in the type of oxidatively modified truncated fatty acyl chain at the *sn-2* position (Figure 1B). They also contain 5-carbon fatty acyl chains with PGPC bearing a ω-carboxyl group, while POVPC bears a polar ω-aldehydic group, both esterified at the *sn-2* position. The ω-aldehydic and ω-carboxylic groups are, respectively, zwitterionic and anionic at neutral pH.

It is considered that OxPCs alone do not arrange into bilayer structures. They aggregate to form micelles between 4 and 9 nm in size in aqueous solution at neutral pH [46]. It is likely that the specific biological action of these oxidized lipids is based on the nature of their short oxidized acyl chain in the *sn-2* position. The critical micellar concentrations of POVPC and PGPC in water are very close, 66.9 and 54.6 μM, respectively [46,47], which could suggest similar coefficients of partition of these lipids to PC membranes. Currently, no data are available on the critical micellar concentration, the size of OxPC micelles at high salt concentration (150 mM), as it is in our experimental conditions. Furthermore, there are no data in the literature on OxPC mixing/demixing temperatures with different lipid classes within a lipid bilayer when physiological concentrations of OxPC are used (30 mol %) depending on the length and degree of unsaturation of their fatty acids, the nature of their polar head or their charge. In the absence of these data, it was a priori not possible for us to know which protocol of lipid mixing is the most convenient for this study.

Nevertheless, we considered it worthwhile to prepare such samples in order to examine the effect of OxPCs on membrane biophysics, as their role is crucial to understand human pathologies related to ageing. This is evidenced by a large body of scientific work describing a significant increase in OxPC levels in the membranes of subjects suffering from various severe pathologies [48,49]. This is why, as mentioned above in the methodology paragraph, we decided to use two mixing protocols for LUV preparation: either a heating/cooling (HC) cycle or a room temperature (RT) preparation. This was carried out in order to partially overcome the lack of prior information on the physochemical properties of OxPCs in PC matrices.

This proved to be a wise methodological decision, as our study showed that POPC LUVs are more ordered at the glycerol backbone than those of PDPC when the mixing was carried out at RT and measured the same day (Figure 6(A1)). This result was expected, as the presence of DHA in *sn-2* of PDPC would have suggested a high degree of disorder and is usually known to make the membrane more fluid [50,51,52]. The impact of the dual sample preparation procedure was relevant not only to the lipid order, but also to vesicle size measurements. In contrast to the results obtained for POPC by RT protocol, when the HC protocol was used, the order parameter of PDPC LUVs was slightly higher than that of POPC ones at the physiological temperature (Figure 6(A2)). These changes in lipid order of the PDPC LUVs might be explained by the higher rate of auto-oxidation of this lipid during the HC cycles [53,54]. At least two factors are associated with a higher probability of oxidation of this lipid. The first is due to the higher temperature during incubation at 60 °C, the second to the longer hydration time (at least 45 min) during LUV preparation in the HC compared to the RT protocol. This is why the samples were measured on the very day they were made. Measurements taken on the following days showed an increase in the lipid order for PDPC membranes, even though all precautions against oxidation had been taken (Supplementary Figure S1). Both OxPCs reduce the lipid order in the membrane bilayer, i.e., they increase the degree of hydration at the glycerol level in both types of PC matrices. This decrease in the order parameter reflects a more mobile molecular microenvironment compared with controls. The presence of short acyl chains in the *sn-2* position of OxPCs thereby facilitates better penetration of water molecules and reorientation of the lipid moieties [55].

The two OxPCs have a different effect on the PC matrix, depending on the nature of the OxPC. PGPC induces a greater decrease in the lipid order than POVPC on both PC matrices. This effect of PGPC is assigned to the more polar functional groups of the short oxidized chains and their greater probability of being oriented in the aqueous solution outside the bilayer [55]. In POPC/OxPC LUVs, there is a correlation between the vesicle size and the membrane lipid order. The lower the lipid order, the smaller the vesicle size. This relationship is not observed for PDPC LUVs, probably due to the existence of two size distributions under the control conditions and only one in the presence of OxPC.

In this context, it is therefore not surprising that the specificity of the membrane physicochemical parameters resulting from the type of lipid mixing protocol can, in turn, modulate the sPLA_2_ enzymatic activity. This is what we show in this study, where we demonstrate that POVPC and PGPC are able to modulate the kinetics of PED6 hydrolysis by sPLA_2_ on PC matrices. The question arises as to whether sPLA_2_ from bee venom is suitable for our study. Indeed, most representatives of the sPLA_2_ family lack specificity to phospholipids with different acyl chains in the *sn-2* position [56]. This is not the case for bee venom sPLA_2_, which shares a high degree of sequence homology with the mammalian group III enzyme sPLA_2_ [57]. sPLA_2_ is able to bind to the membrane surface by non-electrostatic mechanisms [58,59,60]. It is expected that bilayers with a low degree of lipid packing would be more readily accessible for sPLA_2_ binding due to the lower energy needed to push two adjacent lipids apart. Such a correlation between the membrane lipid order and sPLA_2_ activity is observed when comparing membranes in the gel and liquid-disordered phases [12]. Apparently, such an assertion cannot be assigned when one compares two membranes in the liquid-disordered phase. Our results show that enzymatic activity is higher in more ordered monounsaturated POPC membranes than in less ordered polyunsaturated PDPC membranes. It is consistent with the results obtained by Mouchlis and Dennis [61] who reported higher sPLA_2_ activity in POPC compared to PDPC membranes. Their study demonstrates a roughly 10-fold higher affinity of sPLA_2_ to PC with C18:1 and C18:2 at the *sn-2* position compared to polyunsaturated C20:4 and C22:6 acids.

Additionally, OxPC-containing vesicles also exhibited a lower membrane lipid order (Figure 6) and the sPLA_2_ activity was again lower compared to the control membranes.

The inhibitory effect of POVPC and PGPC is supposed to be exerted in two ways: (i) OxPCs decrease sPLA_2_ activity due to a greater affinity to sPLA_2_ than PED6 prior to its hydrolysis of the fatty acid chain in *sn-2*, or (ii) sPLA_2_ only binds to *sn-2* of OxPCs without subsequent hydrolysis. In either case, OxPC could act as a concurrent inhibitor when PED6 is used as a substrate to assess sPLA_2_ activity. Currently, it is established that only three PLA_2_ enzymes (two platelet-activating factor (PAF) acetylhydrolases from group VII and lysosomal PLA_2_ from group XV) are able to hydrolyze truncated diacyl phospholipids [26,27,62]. Therefore, we assume that oxidized lipids POVPC and PGPC, analogous to sphingomyelin (SM), could be physiological inhibitors of sPLA_2_ due to their similar structural features and phosphocholine head groups, when compared to the substrate PC molecules [63,64,65].

OxPCs probably act by competing with PED6 (and/or PC [66]) for binding to the active site of the enzyme. Our results clearly show that POVPC and PGPC decrease both the enzyme rate during burst and steady states of the enzyme reaction (Figure 3). This suggests that POVPC and PGPC engage the enzyme by blocking its active center without hydrolysis of the short-oxidized acid at *sn-2* position. The different functional groups at the end of the oxidized acyl chains of OxPCs have a significant effect not only on molecular packing in the bilayer, but also on sPLA_2_ activity. As mentioned above, the short oxidized tails might migrate towards the water phase [55], thus promoting accumulation of molecular packing defects, thereby favoring an increase in membrane permeability [67]. The truncated chains with an aldehyde group at the end (POVPC) adopt all directions in the interior and exterior of the bilayer while the chains with the carboxyl group at the end (PGPC) are more constrained, sticking out quasi-perpendicular to the bilayer [55]. This structural behavior is in line with our observation that PGPC exhibits a weaker inhibitory effect on sPLA_2_ activity compared to POVPC (Figure 3). We hypothesize that the carboxyacyl chains localized mainly out of the membrane surface sterically hinder the binding of the enzyme to PGPC. Thus, more sPLA_2_ molecules are available for hydrolysis of the substrate as evidenced by the higher FL emission recorded in PGPC-containing vesicles versus POVPC ones. The aldehydoacyl chains that can be oriented both outward and towards the membrane interior exert less steric hindrances for binding of the enzyme to POVPC molecules. Thus, less enzyme molecules are available for the substrate molecules.

The FA nature in all four studied PC molecules also plays an essential role here. On one hand, DHA carbon chains are extremely flexible due to its high degree of molecular freedom while at the same time PDPC is characterized by high electron density near the lipid-water interface [68,69]. On the other hand, *sn-2* carboxyacyl chains of PGPC induce higher lateral diffusion of the membrane lipids compared to *sn-2* aldehydoacyl chains of POVPC [55]. This high flexibility of DHA chains in combination with higher lateral mobility of lipids induced by PGPC molecules might be an additional factor that impedes the binding of the enzyme with the substrate PDPC molecules. POVPC is able to inhibit sPLA_2_ more effectively compared to PGPC in monounsaturated POPC membranes while in polyunsaturated PDPC membranes no significant difference in the effect of two OxPCs is observed. This is an expected result since, as mentioned above, a larger order parameter difference between POVPC- and PGPC-containing membranes is detected in the monounsaturated matrix in comparison to the polyunsaturated one.

An intriguing result is that in samples prepared by RT, the presence of PGPC in polyunsaturated membranes twice doubled the rate of sPLA_2_ activity compared with control vesicles, whereas in monounsaturated membranes, enzyme activity with and without PGPC was almost identical (Table 2).

As the reaction proceeds, the liberated fatty acids could in turn modulate the enzyme activity. Indeed, Figure 3 demonstrates that, at a steady state of the reaction, sPLA_2_ activity is about 2-fold higher for both types of membranes, PDPC/PGPC and POPC/PGPC, compared to the controls. A 109% increase is observed for PDPC/PGPC vesicles and a 74% increase for POPC/PGPC ones (Table 1). Even by using two structural approaches such as changes in vesicle size and lipid order of the membranes, we did not find a correlation between these physicochemical parameters and the high increase in sPLA_2_ activity in the presence of PGPC in both PC matrices in dependence of the protocol of lipid mixing. Although, there is a difference in vesicle size of the membranes in POPC/PGPC mixtures following different protocols of lipid mixing, similar dependence is not seen in PDPC/PGPC mixtures, which apparently could not explain why when lipids are hydrated and mixed at RT contrary to HC cycles, as there is such an increase in the enzyme activity in both lipid matrices. It can be suggested that HC cycles probably change the orientation of the *sn-2* carboxyacyl chains of PGPC relative to the normal membrane, which brings this molecule closer in properties to POVPC. Singh and Ranganathan [70] have shown that, when alone, PGPC forms a micelle (3.5 nm radii micelles with aggregation number 33) and does not form ideal mixtures with the bilayer phospholipids DPPC (C16:0, C16:0 PC; T_m_ = 41 °C) and DOPC (C18:1, C18:1 PC, T_m_ = −16.5 °C). These authors have stated that the formation of mixed vesicles is favored in the gel phase compared to the liquid phase for X_PGPC_ ≤ 0.3 as PGPC is mixed with DPPC at 50 °C, but not at 60 °C, while DOPC does not mix with PGPC in the temperature range from 5° to 60 °C. Therefore, it means that for the studied mixtures, 70/30 POPC/PGPC and PDPC/PGPC, one can expect to have non-mixed components, coexisting separated vesicles and micelles. However, in our experimental conditions, DLS profiles for the hydrodynamic radii of POPC/OxPC and PDPC/OxPC clearly state that there is no evidence for the formation of micelles under our experimental conditions (Figure 7). The size of POPC/PGPC LUVs decreases by about 50% as no peaks are located around 3–10 nm indicative of the coexistence of micelles. In contrast, pure PDPC LUVs show two size distributions whereas PDPC/PGPC mixtures display one symmetric size distribution. It seems that the presence of PGPC even stabilizes the bilayer structure of PDPC leading to 30% vesicles size increase. Our DLS results do not resolve the coexistence of separated LUVs and micelles in the temperature range from 25° to 60 °C, which suggests rather lateral separation of PGPC within the POPC and PDPC membranes or different conformation of the short truncated fatty acid depending on the protocol of lipid mixing.

The difference between our results and those obtained by Singh and Ranganathan [70] for DOPC/PGPC mixtures, stating the existence of micelles at physiologic temperatures, can be assigned to several reasons: high salt concentrations in our experimental conditions versus lack of them, the use of extruded large unilamellar vesicles in our study versus small unilamellar vesicles and finally hetero-acyl phospholipids in this study versus homo-acyl ones in Singh and Ranganathan [70].

## 5. Conclusions

OxPCs are involved in the regulation of enzymatic hydrolytic systems such as PLA_2_ and sphingomyelinase [71]. In this work, we have shown that the truncated oxidized lipids POVPC and PGPC decrease the membrane lipid order at the glycerol level. The effects exerted by these bioactive compounds depend on the different spatial orientations of their more polar shortened acyl chains relative to the degree of unsaturation of the *sn-2* fatty acid chains. PGPC induces a much greater decrease in lipid packing in both lipid matrices compared to POVPC. Phospholipases are overexpressed during inflammation. The elevated levels and activity of these enzymes are considered indicators of cellular ageing and a predisposing factor for various diseases such as cardiovascular and neurodegenerative diseases. The study of the mechanisms responsible for regulating PLA_2_ activity is thus necessary for the development of new therapeutic approaches in the treatment of inflammatory diseases. In our study, using biomimetic systems, we demonstrated that ω-3-containing lipids such as PDPC, which are even more prone to oxidation, are capable of decreasing sPLA_2_ activity in vitro, thus potentially helping to control inflammation.

Overall, POVPC inhibits sPLA_2_ activity whereas PGPC can inhibit or stimulate sPLA_2_ depending on the lipid mixing protocol. This suggests a different lateral organization of PGPC molecules in the two types of PC samples, such as lateral segregation of PGPC within the membrane or a different orientation of truncated oxidized fatty acids in the *sn-2* position. It is likely that sPLA_2_ activity is not directly associated with the hydrolysis of the *sn-2* fatty acid of OxPC [23,26,27,62]. Indeed, our results suggest that the molecular mechanisms by which sPLA_2_ activity is modulated by POVPC or PGPC are complex, and do not appear to be directly or exclusively linked to any single feature of the lipid membrane, such as its membrane lipid order or curvature, etc.

Apparently, the truncated fatty acids of OxPCs can adopt different orientations with respect to the membrane normal, depending on the degree of unsaturation of the surrounding fatty chains, as well as the temperature, and/or pH and ionic strength of the aqueous environment. The interactions between all these parameters thus generate unstable systems that are sensitive to minor changes in experimental conditions. These uncertainties may explain the inconsistencies regarding sPLA_2_ activity on mono- or polyunsaturated matrices containing truncated oxidized lipids as a function of a lipid mixing protocol. Difficulties in experimental reproducibility certainly play a part in these differences between studies. A more precise identification of the structural and organizational diversity of these oxidized lipids within membranes is necessary if we want to approximate physiological conditions, particularly the effects of OxPCs on enzymes and signaling proteins. These data would be even more important as OxPCs are described as exerting a significant pro-inflammatory effect, particularly via their interactions with receptors of the innate immune system, such as Toll-like receptors [72,73], scavenger receptors [73,74], natural antibodies and other effectors [75]. Nevertheless, several studies have shown, conversely, that OxPCs can attenuate inflammatory responses by inhibiting Toll-like receptor activation as well as phagocytosis [31,76]. Such discrepancies are not yet resolved [76]. Our results support an anti-inflammatory effect of POVPC, since it is intrinsically capable of inhibiting sPLA_2_ activity, this inhibition being independent of the degree of fatty acid unsaturation in the membrane and the lipid mixing protocol. This is not the case for PGPC, which, depending on the mixing protocol, may have pro- or anti-inflammatory activity, highlighting the importance of methodological issues in this type of study.

There is growing evidence that oxidized phospholipids play a key role in the development of various chronic diseases, including cardiovascular, neurodegenerative and neoplastic diseases. The use of oxidized phospholipid molecules is a promising tool to better understand the biological functions of complex lipid systems such as biological membranes, and to reveal the intimate mechanisms underlying the onset and development of oxidation-related pathologies.

## Figures and Tables

**Table 1:** Summary of the relative changes ΔFL/FL_PC_ of steady state of sPLA_2_ hydrolysis in PC/OxPC mixtures. RT—room temperature protocol for lipid mixing; HC—heating/cooling cycles for lipid mixing.

ΔFL/FL_PC_ Values of sPLA_2_ Hydrolysis (%) at Steady State			
POPC+	ΔFL/FL	PDPC+	ΔFL/FL
30% PGPC (RT)	+74.4	30% PGPC (RT)	+109.1
30% PGPC (HC)	−43.1	30% PGPC (HC)	−63.0
30% POVPC (RT)	−59.3	30% POVPC (RT)	−20.3
30% POVPC (HC)	−77.7	30% POVPC (HC)	−78.8

**Table 2:** Parameters of the linear kinetics calculated by fitting sPLA_2_ fluorogenic assay data by [*FL = F*_530_*/F*_530*, initial*_ − *1*] = *a* + *bt*. RT—room temperature protocol for lipid mixing; HC—heating/cooling cycles for lipid mixing.

Composition	FL = F_530_/F_530, initial_ − 1 at *t* = 0 min; *a*	PLA_2_ Reaction Rate, *b*, min^−1^
POPC (RT)	0.073 ± 0.007	0.060 ± 0.003
POPC + 30% POVPC (RT)	0.026 ± 0.003	0.023 ± 0.002
POPC + 30% PGPC (RT)	0.085 ± 0.003	0.053 ± 0.002
POPC (HC)	0.179 ± 0.012	0.080 ± 0.007
POPC + 30% POVPC (HC)	0.015 ± 0.001	0.012 ± 7.79 × 10^−4^
POPC + 30% PGPC (HC)	0.059 ± 0.003	0.021 ± 0.002
PDPC (RT)	0.046 ± 0.004	0.033 ± 0.002
PDPC + 30% POVPC (RT)	0.036 ± 0.001	0.026 ± 8.55 × 10^−4^
PDPC + 30% PGPC (RT)	0.106 ± 0.004	0.063 ± 0.003
PDPC (HC)	0.049 ± 0.003	0.039 ± 0.002
PDPC + 30% POVPC (HC)	0.000 ± 0.001	0.007 ± 8.61 × 10^−4^
PDPC + 30% PGPC (HC)	0.017 ± 0.001	0.005 ± 6.63 × 10^−4^

## Data Availability

Data are available from the corresponding author upon reasonable request and are deposited on the institution website of the corresponding author (http://biomed.bas.bg/bg/, accessed on 13 May 2023).

## Supplementary Materials

The following supporting information can be downloaded at: https://www.mdpi.com/article/10.3390/ijms241311166/s1.
