# Supplementary material for: Impact of Truncated Oxidized Phosphatidylcholines on Phospholipase A2 Activity in Mono- and Polyunsaturated Biomimetic Vesicles
Source: Int J Mol Sci. 2023 Jul 6;24(13):11166. doi: 10.3390/ijms241311166 (PMC10342369; doi:10.3390/ijms241311166)
Supplement: Supplementary file 1 [file ijms-24-11166-s001.zip › ijms-2440016-supplementary.pdf]

Supplementary Data

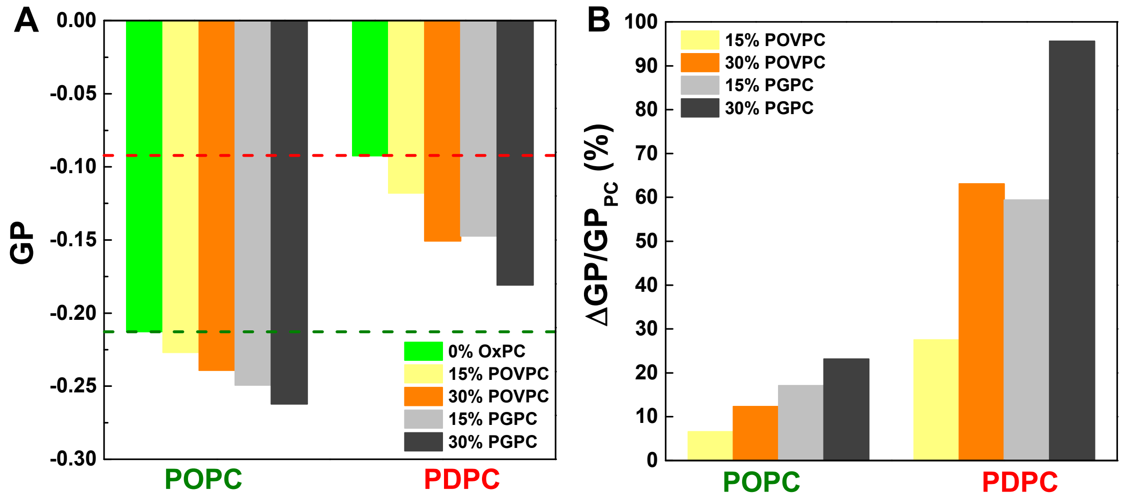

Figure S1. Effect of the degree of unsaturation at *sn-2* position of PC and OxPC (POVPC or PGPC) on lipid packing of PC membranes in L_d phase measured by Laurdan experiments:

(A) Laurdan GP in control POPC (green dashed line) and PDPC (red dashed line) LUV and in binary OxPC-containing (15 and 30 mol %) ones with lipid/Laurdan ratio 200:1 mol/mol **hydrated with heating/cooling cycles and <u>measured the next day</u>.** The lines are added only for clarity. GP values correspond to the lipid bilayer packing. The data represent means at 37°C from measuring the GP values as a function of temperature (20 – 60°C). Error bars are not shown as they were too small to be displayed;

(B) $\Delta GP/GP_{PC}$ (%) quantifies the reduction in membrane lipid packing in presence of OxPC in comparison to control vesicles without OxPC. ΔGP is defined as the difference between GP values for binary OxPC-containing vesicles and control PC ones (GP_{PC}). The explanation for calculating these changes comes from the fact that control PC membranes show different GP values.

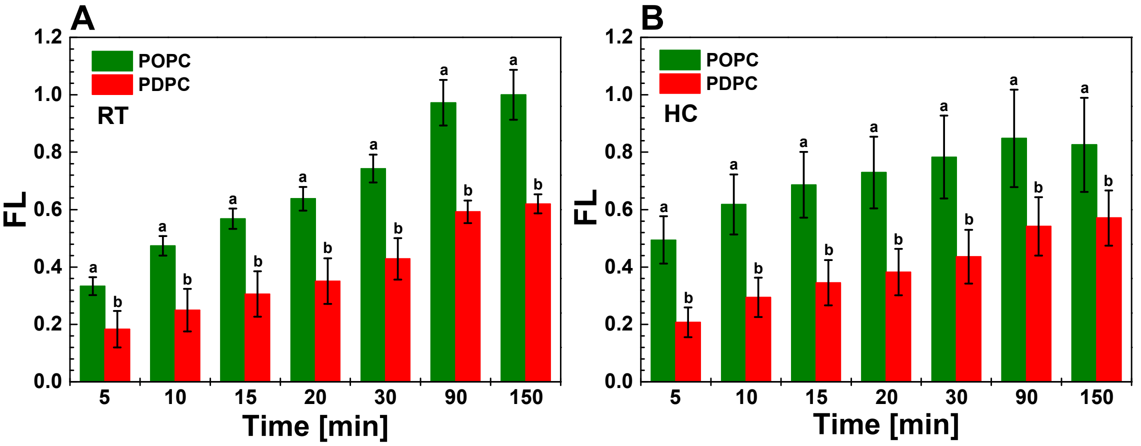

Figure S2. Hydrolysis of PED6 incorporated in control POPC and PDPC vesicles hydrated at room temperature without heating and cooling, RT, (A) and with heating and cooling cycles, HC, (B). FL represents the ratio of fluorescence intensity of PED6-labeled vesicles at time *t*, and fluorescence intensity at 530 nm before enzyme addition minus 1, $FL = F_{530}/F_{530,\ initial} - 1$. It is compared at different times (5, 10, 15, 20, 30, 90 and 150 min) of

the fluorogenic assay. Error bars express standard deviations (n=12). One-Way ANOVA method for means comparison was performed. The data is drawn from a normally distributed population, and the population means are significantly different at the 0.05 level, indicated by different letters above bars.

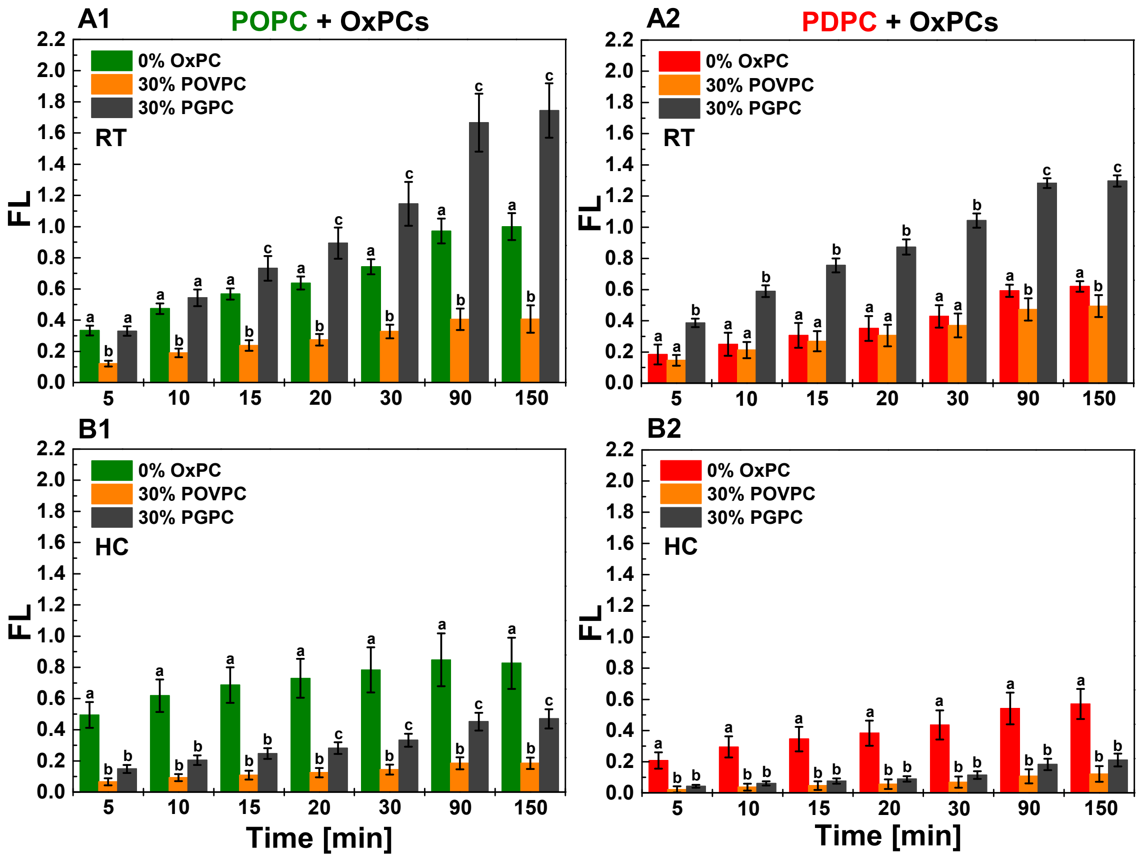

Figure S3. Hydrolysis of PED6 incorporated in POPC (1) and PDPC (2) vesicles containing OxPC (POVPC or PGPC) at 0 (controls) and 30 mol % by $sPLA_2$. Samples are hydrated at room temperature without heating and cooling, RT, (A) and with heating/cooling cycles, HC, (B). FL represents the ratio of fluorescence intensity of PED6-labeled vesicles at time *t*, and fluorescence intensity at 530 nm before enzyme addition minus 1, $FL = F_{530}/F_{530,\ initial} - 1$. It is compared at different times (5, 10, 15, 20, 30, 90 and 150 min) of the fluorogenic assay. Error bars express standard deviations (n=12). One-Way ANOVA method for means comparison was performed. The data is drawn from a normally distributed population. Different letters above bars indicate the statistically significant differences, at time *t*, based on Tukey test with $p < 0.05$.
